# Community exposures among Colorado adults who tested positive for SARS-CoV-2 –A case-control study, March-December 2021

**DOI:** 10.1371/journal.pone.0282422

**Published:** 2023-03-02

**Authors:** Alice E. White, Amanda D. Tran, Michelle R. Torok, Rachel H. Jervis, Bernadette A. Albanese, Andrea G. Buchwald, Emma Schmoll, Ginger Stringer, Rachel K. Herlihy, Elaine J. Scallan Walter

**Affiliations:** 1 Department of Epidemiology, Colorado School of Public Health, Aurora, Colorado, United States of America; 2 Communicable Disease Branch Division of Disease Control and Public Health Response, Colorado Department of Public Health and Environment, Denver, Colorado, United States of America; 3 Tri-County Health Department, Greenwood Village, Colorado, United States of America; 4 Center for Innovative Design and Analysis, Colorado School of Public Health, Aurora, Colorado, United States of America; University of Ilorin, NIGERIA

## Abstract

**Objectives:**

Severe acute respiratory syndrome coronavirus 2 (SARS-CoV-2) infection, which causes coronavirus disease 2019 (COVID-19), is spread primarily through exposure to respiratory droplets from close contact with an infected person. To inform prevention measures, we conducted a case-control study among Colorado adults to assess the risk of SARS-CoV-2 infection from community exposures.

**Methods:**

Cases were symptomatic Colorado adults (aged ≥18 years) with a positive SARS-CoV-2 test by reverse transcription-polymerase chain reaction (RT-PCR) reported to Colorado’s COVID-19 surveillance system. From March 16 to December 23, 2021, cases were randomly selected from surveillance data ≤12 days after their specimen collection date. Cases were matched on age, zip code (urban areas) or region (rural/frontier areas), and specimen collection date with controls randomly selected among persons with a reported negative SARS-CoV-2 test result. Data on close contact and community exposures were obtained from surveillance and a survey administered online.

**Results:**

The most common exposure locations among all cases and controls were place of employment, social events, or gatherings and the most frequently reported exposure relationship was co-worker or friend. Cases were more likely than controls to work outside the home (adjusted odds ratio (aOR) 1.18, 95% confidence interval (CI): 1.09–1.28) in industries and occupations related to accommodation and food services, retail sales, and construction. Cases were also more likely than controls to report contact with a non-household member with confirmed or suspected COVID-19 (aOR 1.16, 95% CI: 1.06–1.27).

**Conclusions:**

Understanding the settings and activities associated with a higher risk of SARS-CoV-2 infection is essential for informing prevention measures aimed at reducing the transmission of SARS-CoV-2 and other respiratory diseases. These findings emphasize the risk of community exposure to infected persons and the need for workplace precautions in preventing ongoing transmission.

## Introduction

The coronavirus disease 2019 (COVID-19) pandemic has had a profound impact on the health and wellbeing of people and economies around the globe. Early in the pandemic, many countries implemented stay-at-home orders, restaurant closures, international travel restrictions, and other public health interventions to reduce the transmission of severe acute respiratory syndrome coronavirus 2 (SARS-CoV-2), the virus that causes COVID-19 disease. SARS-CoV-2 is spread by respiratory or aerosolized droplets through close contact with symptomatic and asymptomatic persons, resulting in rapid transmission in the community. Close contact with a person with confirmed or suspected COVID-19 is a dominant and well-documented source of spread [1]. To mitigate rapid community spread, governments and public health agencies instituted widespread and sweeping control measures, such as mask mandates and stay-at-home orders. However, assessing the contribution of community exposures is challenging because of the nature of person-to-person SARS-CoV-2 transmission.

In Colorado, a study of persons with laboratory-confirmed COVID-19 in March 2020 reported that the most common activities two weeks prior to symptom onset included attending gatherings of >10 persons, travelling domestically, working in a healthcare setting, visiting a healthcare setting not as a health care worker, and using public transportation [1]. However, this study was limited by the absence of a comparison group. In July 2020, the Centers for Disease Control and Prevention (CDC) conducted a case-control study in 11 outpatient facilities in the United States to evaluate community and close contact exposures. The study found that adults with positive SARS-CoV-2 tests were twice as likely to report dining at a restaurant than those with negative SARS-CoV-2 tests and less likely to report working from home or teleworking [2, 3].

Studies characterizing how community transmission occurred are needed to better target control measures and public health interventions to mitigate the spread of SARS-CoV-2 and other respiratory infections. Here, we present findings from a case-control study that assessed close contact and community exposures associated with SARS-CoV-2 infection among Colorado adults during 2021.

## Methods

Cases were symptomatic Colorado adults (aged ≥18 years) with SARS-CoV-2 infection confirmed by reverse transcription-polymerase chain reaction (RT-PCR). From March 16 to December 23, 2021, cases were randomly sampled ≤12 days after their specimen collection date from among those with a completed case investigation interview in Colorado’s COVID-19 surveillance system. Controls were randomly selected from persons with a RT-PCR-confirmed negative SARS-CoV-2 test result reported to Colorado’s Electronic Laboratory Reporting (ELR) system. Cases were individually matched to up to 20 controls on age (±10 years), zip code (urban areas) or region (rural/frontier areas), and specimen collection date (3–7-day period corresponding to sample selection).

An online survey developed in Research Electronic Data Capture (REDCap) collected data from cases and controls about travel (state, national, and international), contact with anyone with confirmed or suspected COVID-19, and community activities in the 14 days before illness onset (cases) or specimen collection (controls). Community activities included going in-person to a bar or club; church, religious, or spiritual gathering; grocery or retail shopping; gym or fitness center; healthcare setting; public transportation; restaurant, café, or coffee shop; dining at a restaurant indoors; salon, spa, or barber; social event or gathering; sports or sporting events. Data on COVID-19-related symptoms, working or volunteering outside the home (including occupation and industry type), and demographic characteristics (sex, age, race/ethnicity) were available from Colorado’s COVID-19 surveillance system for cases; these fields were included in the online survey for controls.

Both cases and controls were sent a text message inviting them to complete the online survey; during March-April 2021, trained interviewers also attempted to contact non-responders by telephone if they did not complete the online survey. One contact attempt was made for cases and up to two contact attempts for controls. The online survey was available in English and Spanish, and interviewers had access to a language line interpreter. In order to focus on community exposures, cases and controls were excluded if they reported living in an institution or close contact with a household member with confirmed or suspected COVID-19. Other exclusion criteria included duplicate or missing telephone number or missing zip code; symptom onset date >7 days from the specimen collection date (cases); a prior positive COVID-19 test result (controls); or failed data quality checks (e.g., straight-lined answers, majority of answers unknown, personal identifying information provided in online survey that was inconsistent with ELR). Self-reported COVID-19 vaccination (number of doses, type) was recorded after May 13, 2021; before then, participants reporting any vaccination were excluded.

Occupation and industry types were assigned using the National Institute for Occupational Safety and Health (NIOSH) Industry and Occupation Computerized Coding System (NIOCCS), a web-based software tool designed to translate industry and occupation text to standardized industry and occupation codes. Counties were classified as urban or rural/frontier based on Colorado census designations.

An unmatched analysis using logistic regression assessed differences between cases and controls adjusting for matched criteria (age, geographical region, specimen collection period) and potential confounders selected a priori. A matched analysis using conditional logistic regression was also performed. A separate model restricted the analysis to symptomatic controls to make the control group more comparable to cases and to adjust for potential differences in behaviors and recall between asymptomatic and symptomatic persons. Statistical analyses were conducted using SAS software (version 9.4; SAS Institute). The Colorado Multiple Institutional Review Board determined this study to be public health surveillance and not human subjects research and therefore exempt from full approval and requirements for informed consent.

## Results

**Fig 1:**
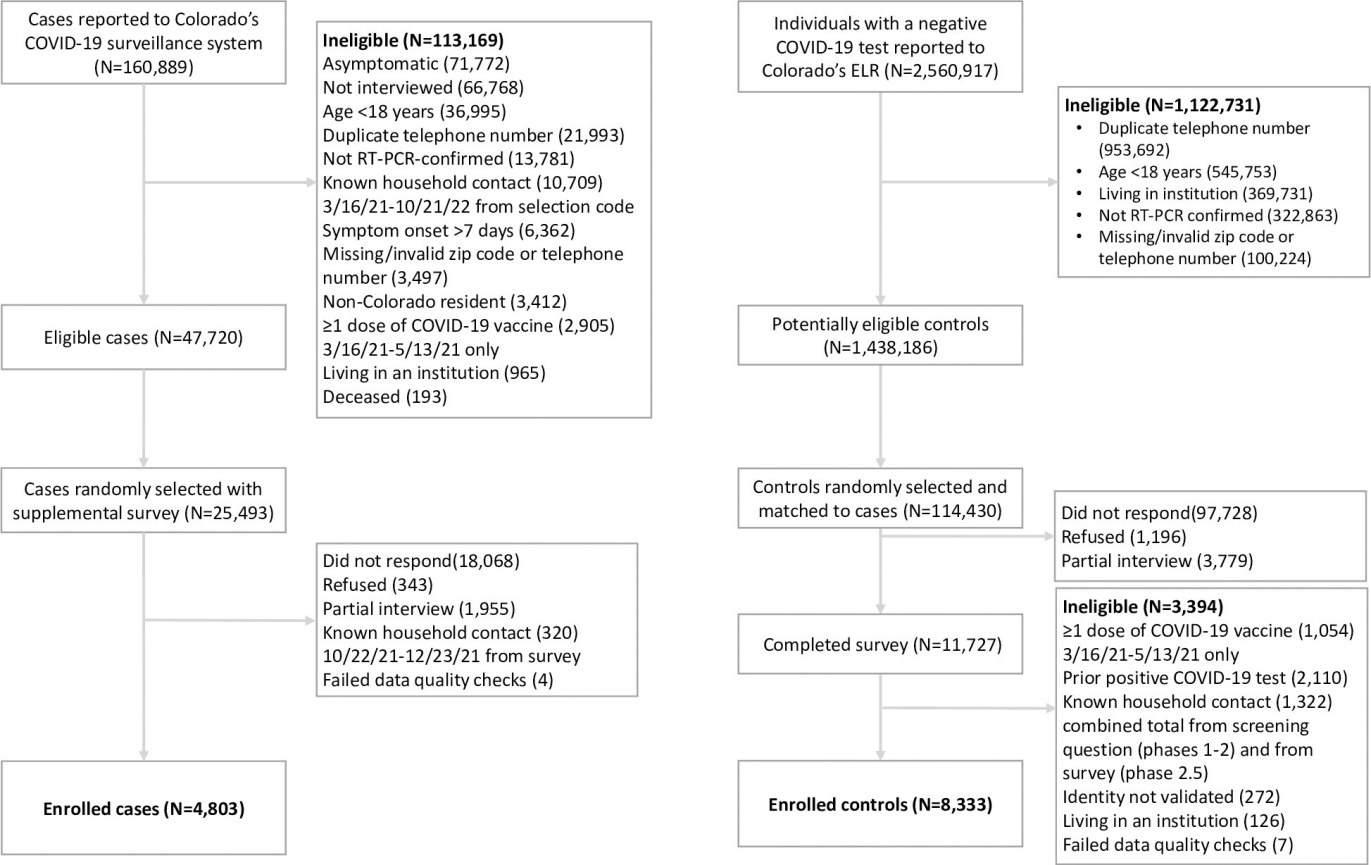
Eligibility and enrollment cases (persons with positive SARS-CoV-2 test results) and controls (persons with negative SARS-CoV-2 test results) among Colorado adults aged ≥18 years–specimen collection dates from March 16 to Deceber 23, 2021.

This study included 4,803 cases and 8,333 controls. Of the eligible cases randomly selected for the supplemental interview (25,493), 4,803 were enrolled (response rate 19%). Of the potentially eligible controls randomly selected and matched to cases (114,430), 8,333 were enrolled (response rate 7%) (Fig 1). Compared to the population of all eligible and randomly selected cases, enrolled cases were older, more likely to identify as female, less likely to identify as Black or Hispanic, and more likely to be fully vaccinated. Compared to the population of all eligible and randomly selected controls, enrolled controls were older, less likely to identify as Black or Hispanic, and less likely to reside in rural or frontier counties. There were more enrolled cases in the 18–29 years age groups (20% versus 10%) and in rural/frontier counties (19% versus 16%) compared to enrolled controls. Cases were more likely to identify as Hispanic (17% versus 9%). Cases were more likely to be unvaccinated than controls (29% versus 5%) (Table 1). Most controls (4,987; 60%) reported COVID-19-like symptoms in the 14 days before their specimen collection date.

**Table 1:** Characteristics of symptomatic Colorado adults aged ≥18 years with confirmed COVID-19 (enrolled cases) compared to persons testing negative for COVID-19 (enrolled controls) and the populations from which they were selected–specimen collection dates from March 16 to December 23, 2021.

Characteristic	Cases (n = 4,803)		Case Pool^a^ (n = 25,493)		Controls (n = 8,333)		Control Pool (n = 114,430)	
	N	%	N	%	N	%	N	%
**Age group (years)**								
18–29	949	19.8	6,758	26.5	798	9.6	24,480	21.4
30–39	1,119	23.3	6,058	23.8	1,962	23.5	30,227	26.4
40–49	992	20.7	4,615	18.1	1,838	22.1	23,160	20.2
50–59	809	16.9	3,519	13.8	1,693	20.3	18,645	16.3
≥60	934	19.5	4,543	17.8	2,042	24.5	17,918	15.7
**Age, mean (SD)**	44.1 (15.3)		42.3 (16.3)		47.8 (14.2)		42.6 (14.7)	
**Gender (missing = 111)**			(missing = 6)				NA^c^	
Female	2,894	60.3	13,674	53.7	5,359	65.2	NA	
Male	1,846	38.5	11,567	45.4	2,798	34.0	NA	
Another	60	1.3	246	1.0	68	0.8	NA	
**Race/Ethnicity (missing = 1,309)**			(missing = 3,718)				(missing = 41,688)	
Black, non-Hispanic	113	2.7	980	4.5	140	1.8	2,995	4.1
Hispanic	706	16.7	5,232	24.0	693	9.1	7,650	10.5
Other, non-Hispanic	150	3.6	863	4.0	306	4.0	12,468	17.1
White, non-Hispanic	3,254	77.1	14,700	67.5	6,465	85.0	49,629	68.2
**Geographic location**								
Rural or Frontier	904	18.8	5,003	19.6	1,327	15.9	21,508	18.8
Urban	3,899	81.2	20,490	80.4	7,006	84.1	92,922	81.2
**Self-reported vaccination**^d^ **(missing = 979)**			(missing = 3,865)					
Fully vaccinated	2,437	68.1	8,952	45.0	6,797	91.9	NA^c^	
Partially vaccinated	105	2.9	738	3.7	200	2.7	NA	
Unvaccinated	1,039	29.0	10,215	51.3	399	5.4	NA	

NA = Not available

^a^ Cases reported to Colorado’s COVID-19 surveillance system who met study eligibility criteria and were randomly selected to be sent the online survey

^b^ Individuals with a negative COVID-19 test reported to Colorado’s Electronic Laboratory Reporting system who met initial eligibility criteria who were matched to cases and randomly selected to be sent the online survey

^c^ Gender and vaccination not available from electronic laboratory reports for controls

^d^ Individuals with any vaccine dose were excluded from the study March 16 –May 13 2021 (535 cases, 645 controls). Individuals were considered fully vaccinated if they received two doses of the two-dose mRNA series (Pfizer, Moderna) or one dose of a single-dose vaccine (Johnson & Johnson) at least 14 days before COVID testing

**Fig 2:**
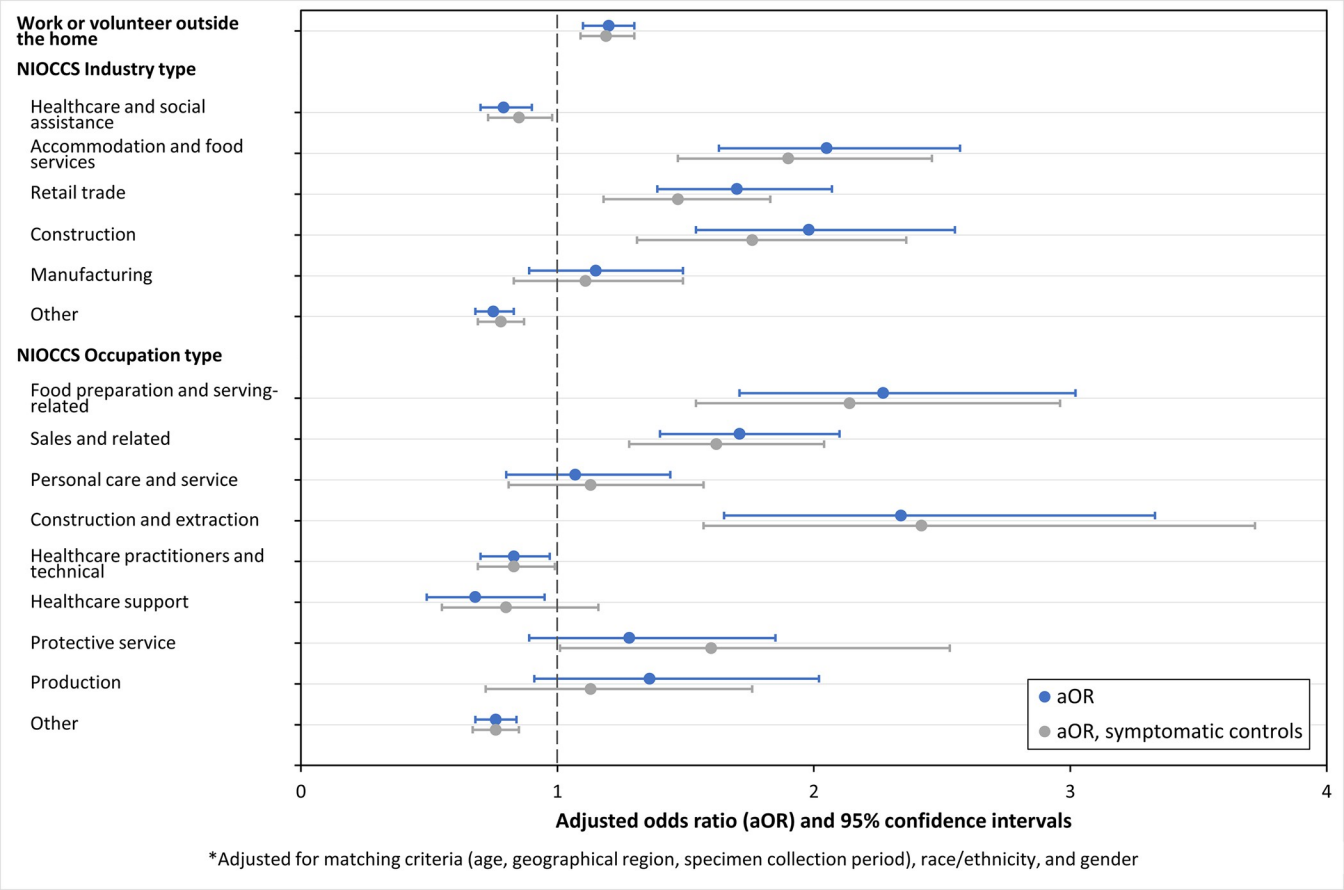
Adjusted odds ratio (aOR)* and 95% confidence intervals for occupations and industries associated with confirmed COVID-19 among symptomatic Colorado adults aged ≥18 years–specimen collection dates from March 16 to December 23, 2021. * Adjusted for matching criteria (age, geographical region, specimen collection period), race/ethnicity, and gender.

Most cases (3,115; 65%) were matched to at least one control. Matched and unmatched analyses produced similar findings; to include all cases, unmatched results are presented here. Cases were more likely than controls to work or volunteer outside the home (aOR 1.18, 95% CI: 1.09–1.28) (Table 2, Fig 2). More cases reported working in industries related to accommodation and food services (aOR 2.05, 95% CI: 1.63–2.57), retail trade (aOR 1.70, 95% CI: 1.39–2.07), and construction (aOR 1.98, 95% CI: 1.54–2.55), whereas controls were more likely to work in healthcare and social assistance (aOR 0.79, 95% CI: 0.70–0.90) (Table 2). In the analysis with only symptomatic controls, these findings remained consistent (Fig 2).

**Table 2:** Community and workplace exposures of symptomatic Colorado adults aged ≥18 years with confirmed COVID-19 compared to persons testing negative for COVID-19 –specimen collection dates from March 16 to December 23, 2021.

	Total Study Population			Persons with no known close contact exposure
	Positive case (n = 4,803), n (%)	Negative control (n = 8,333), n (%)	Odds Ratio (95% CI)^a^	Odds Ratio (95% CI)^a^
**Work or volunteer outside the home**	2,857 (59.7)	4,337 (52.3)	1.18 (1.09–1.28)	1.24 (1.13–1.36)
NIOCCS Industry type^b^				
Healthcare and social assistance	534 (18.7)	1,075 (24.8)	0.79 (0.70–0.90)	0.72 (0.62–0.84)
Accommodation and food services	253 (8.9)	154 (3.6)	2.05 (1.63–2.57)	2.17 (1.65–2.86)
Retail Trade	290 (10.2)	235 (5.4)	1.70 (1.39–2.07)	1.70 (1.35–2.15)
Construction	188 (6.6)	130 (3.0)	1.98 (1.54–2.55)	1.87 (1.40–2.51)
Manufacturing	125 (4.4)	161 (3.7)	1.15 (0.89–1.49)	1.23 (0.91–1.67)
Other	1,467 (51.4)	2,582 (59.5)	0.75 (0.68–0.83)	0.78 (0.69–0.89)
NIOCCS Occupation type^b^				
Food preparation and serving-related	163 (5.7)	89 (2.1)	2.27 (1.71–3.02)	2.48 (1.74–3.54)
Sales and related	271 (9.5)	220 (5.1)	1.71 (1.40–2.10)	1.76 (1.39–2.23)
Personal care and service	84 (2.9)	136 (3.1)	1.07 (0.80–1.44)	1.13 (0.80–1.60)
Construction and extraction	109 (3.8)	60 (1.4)	2.34 (1.65–3.33)	2.25 (1.51–3.36)
Healthcare practitioners and technical	291 (10.2)	607 (14.0)	0.83 (0.70–0.97)	0.77 (0.63–0.94)
Healthcare support	61 (2.1)	131 (3.0)	0.68 (0.49–0.95)	0.60 (0.40–0.91)
Protective service	67 (2.4)	74 (1.7)	1.28 (0.89–1.85)	1.30 (0.84–2.01)
Production	66 (2.3)	59 (1.4)	1.36 (0.91–2.02)	1.41 (0.86–2.29)
Other	1,745 (61.2)	2,961 (68.3)	0.76 (0.68–0.84)	0.76 (0.67–0.86)
**Close contact with a person with confirmed or suspected COVID-19 (non-household member)**	1,283 (26.8)	1,902 (22.9)	1.16 (1.06–1.27)	-
Location of exposure				
Place of employment	61 (41.5)	41 (42.7)	0.90 (0.76–1.06)	-
Social event or gathering	35 (23.8)	18 (18.8)	1.05 (0.89–1.25)	-
In house but not living in household	127 (9.9)	131 (6.9)	1.44 (1.09–1.90)	
Daycare or school	86 (6.7)	145 (7.6)	0.86 (0.63–1.16)	
Healthcare facility	76 (5.9)	153 (8.0)	0.75 (0.55–1.02)	
Travel	73 (5.7)	85 (4.5)	1.37 (0.96–1.94)	
Other	171 (13.3)	263 (13.8)	0.94 (0.75–1.17)	-
Relationship to close contact				
Co-worker	368 (28.7)	518 (27.2)	0.95 (0.80–1.13)	-
Friend	349 (27.2)	487 (25.6)	1.08 (0.91–1.29)	-
Family	301 (23.5)	325 (17.1)	1.66 (1.37–2.01)	-
Other	233 (18.2)	497 (26.1)	0.61 (0.51–0.74)	-
**Travel**				
International travel	99 (2.1)	301 (3.6)	0.60 (0.47–0.77)	0.56 (0.43–0.74)
Travel within the U.S, outside of Colorado	989 (20.6)	1,816 (21.8)	0.99 (0.90–1.09)	0.97 (0.87–1.08)
Travel within Colorado	866 (18.0)	1,755 (21.1)	0.78 (0.70–0.86)	0.77 (0.69–0.87)
**Community exposures**				
Grocery or retail shopping	3,980 (84.2)	7,586 (91.5)	0.49 (0.44–0.56)	0.49 (0.43–0.57)
Restaurant, café, or coffee shop	2,670 (55.9)	5,211 (62.8)	0.82 (0.75–0.88)	0.83 (0.75–0.91)
Indoors at a restaurant, café, or coffee shop	2,230 (87.6)	4,311 (85.9)	1.19 (1.02–1.39)	1.27 (1.06–1.51)
Healthcare setting	1,102 (23.0)	2,570 (30.9)	0.73 (0.67–0.80)	0.70 (0.63–0.77)
Gym or fitness center	742 (15.5)	1,368 (16.5)	0.93 (0.84–1.03)	0.98 (0.87–1.11)
Bar or club	860 (18.0)	1,267 (15.3)	1.16 (1.05–1.29)	1.21 (1.08–1.37)
Salon, spa, or barber	16.4 (782)	1,844 (22.3)	0.74 (0.67–0.81)	0.72 (0.64–0.80)
Church, religious, or spiritual gathering	515 (10.8)	856 (10.3)	1.19 (1.05–1.35)	1.13 (0.98–1.31)
Sports or sporting event, including snow sports	721 (15.1)	1,275 (15.3)	0.96 (0.86–1.07)	1.03 (0.92–1.17)
Social event or gathering	2,812 (58.7)	5,174 (62.2)	0.93 (0.85–1.00)	0.91 (0.83–1.00)
Public transportation	528 (11.0)	1,112 (13.4)	0.83 (0.74–0.94)	0.82 (0.72–0.94)

^a^ Adjusted for matching criteria (age, geographical region, specimen collection period), race/ethnicity, gender

^b^ Industry and occupation types among those who reported working outside the home. Assigned using the NIOSH Industry and Occupation Computerized Coding System

^c^ Community exposures do not include activities undertaken as part of employment

**Fig 3:**
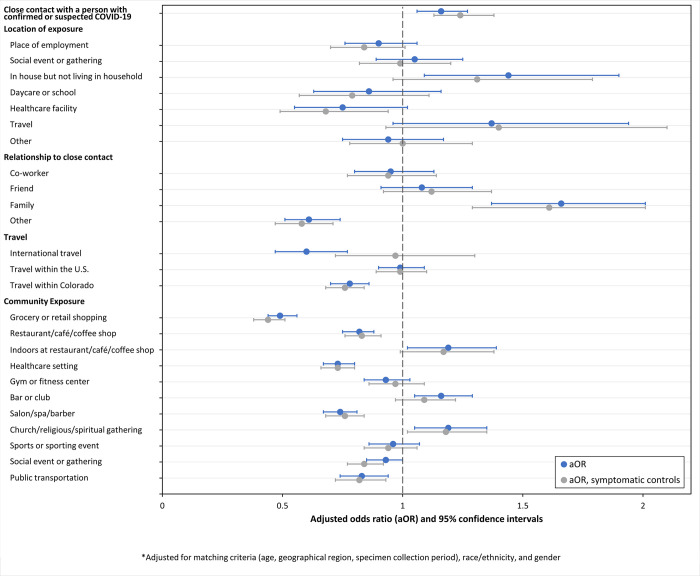
Adjusted odds ratio (aOR)* and 95% confidence intervals for exposures associated with confirmed COVID-19 among symptomatic Colorado adults aged ≥18 years–specimen collection dates from March 16 to December 23, 2021. *Adjusted for matching criteria (age, geographical region, specimen collection period), race/ethnicity, and gender.

Cases were more likely than controls to report contact with a non-household member with confirmed or suspected COVID-19 (aOR 1.16, 95% CI: 1.06–1.27). The most common exposure locations reported by both cases and controls were place of employment and social events or gathering. The most frequently reported exposure relationship was co-worker or friend. Controls were more likely than cases to report travel, grocery or retail shopping, restaurant, café, or coffee shop, healthcare setting, salon, spa, or barber, and public transportation. Cases were more likely than controls to report dining indoors at a restaurant, café, or coffee shop (aOR 1.19, 95% CI: 1.02–1.39), going to a bar or club (aOR 1.16, 95% CI: 1.05–1.29), and attending a church, religious, or spiritual gathering (aOR 1.19, 95% CI: 1.05–1.35). Findings were consistent among cases and controls with no known close contact exposure (Table 2). In the analysis with only symptomatic controls, international travel, dining indoors at a restaurant, café, or coffee shop, and bar or club were no longer statistically significant (Fig 3).

## Discussion

In this case-control study in Colorado, working or volunteering outside the home and close contact with a non-household member with COVID-19 were risk factors for COVID-19. Moreover, among cases who reported close contact with a non-household member with COVID-19, the most frequently reported location was the workplace and the most frequently reported relationship was a co-worker. These findings emphasize the importance of workplace precautions, including sick leave, improved ventilation, reduced crowding, and vaccination, in preventing the ongoing transmission of SARS-CoV-2 and other respiratory viruses [4].

Cases were more likely to leave their home to work as opposed to working from home or teleworking. This finding is congruent with a study that found cases with COVID-19 were more likely to report exclusively working in an office or school setting when compared to controls and another that found that working on-site was positively associated with SARS-CoV-2 infection [3, 5]. As more businesses shift back to in-person work, providing workers with the option to work from home or telework when possible continues to be an important consideration for reducing SARS-CoV-2 transmission. Notably, cases in this study were more likely to work in industries and occupations that cannot be performed remotely, such as accommodation and food services, retail sales, and construction.

Close contact with a non-household member with confirmed or suspected COVID-19 was more frequently reported among cases. This finding is similar to previous reports [1, 2] and underscores the importance of close contact exposure as a driver of the pandemic. In a study of predictors of SARS-CoV-2 infection following a high-risk exposure, mask usage was shown to be protective for people who reported contact with a non-household member known or suspected to be infected with SARS-CoV-2 ≤ two weeks before testing [6]. Businesses should adhere to CDC, state, and local guidance for workplaces to minimize risk of exposure for their employees and customers.

While cases were more likely to work in food service industries such as restaurants, cafés, or coffee shops, reported non-work related exposures in those settings were not associated with infection. This may be related to the timing of this study, which was conducted when some communities encouraged people to return to restaurant dining, and facilities may have been implementing measures to limit dining capacity, use open-air spaces (consistent with our finding cases were more likely to dine indoors), or improve ventilation, although implementation of these guidelines varied by facility and jurisdiction. Evidence from other case-control studies is mixed. While a case-control study in United States conducted in 2020 found that adults with positive SARS-CoV-2 tests were twice as likely to report dining at a restaurant than those with negative SARS-CoV-2 tests, a case-control study in Portugal in 2020 did not find a positive association between going to a restaurant and SARS-CoV-2 infection [5]. Similar to our study, neither study found an increased risk of SARS-CoV-2 associated with other community activities such as going to grocery stores, beauty salons, bars, or the use of public transportation.

The findings in this report are subject to several limitations. First, the COVID-19 positive and negative persons included in this study could differ from those in the general population. Enrolled cases and controls differed from the eligible population from which they were sampled; in particular, cases were more likely to be vaccinated. This study only included cases reported to Colorado’s COVID-19 surveillance system who completed both the case investigation interview and responded to the survey. There was a higher proportion of healthcare workers among controls, who are subject to more frequent testing and may be more willing to answer a survey. Moreover, controls were more likely to travel and have certain community exposures; this may be because controls were more likely to seek testing prior to travel or other planned exposures and were not necessarily representative of the general population who did not have COVID-19 infection. Second, recruitment was limited to symptomatic cases, whose behavior may differ from asymptomatic cases. To control for any resulting bias, the adjusted analysis was restricted to symptomatic controls, which may have led to overmatching because the symptomatic controls likely had other respiratory viruses linked to similar risk behaviors associated with risk for SARS-CoV-2 infection. Third, both cases and controls were aware of their test results, which may have led to social desirability bias; in particular, cases may have underreported exposures.

Knowledge on the settings and activities associated with a higher risk of SARS-CoV-2 transmission is essential for informing prevention measures aimed at reducing the transmission of COVID-19. These findings emphasize the risk of community exposure to persons with COVID-19 and the need for workplace precautions in preventing the ongoing transmission of SARS-CoV-2.
